# Health Tract, No. 88

**Published:** 1865

**Authors:** 


### DEATH OF DEBT.

"This is the happiest day I have had in twenty years! I feel free," said one of the greatest ornaments of the District Court of the United States, to his sunny-faced child.

"What makes you feel so happy, papa?" asked the little girl.

"I am out of debt! I have paid the last dollar I owed in the world, and have been laboring with all my might for twenty years to work myself out of the miserable slavery."

On the first day of April, 1862, Mrs. F——, of S——, was awakened by a tap at the door early in the morning, her husband being in the army. She spoke a word to one of her children, and was a corpse! She thought it was the landlord come for his rent, and knowing she had not a dollar in the house, expected to be turned into the street.

The spacious halls of that fine mansion in a fashionable street in Boston, were lighted up for a gay party. The wife and two daughters had sent out their cards of invitation, and a joyous reünion of friends was anticipated. Already had they began to assemble. At that very moment, the husband and father, having murdered his inexorable creditor, was burning to ashes the dead body of the unfortunate Parkman. It was not meant to intimate that debt would die; that the happy time would ever arrive when pecuniary obligation would become extinct; but that debt brought death, literal death, sometimes, and sometimes, what is far worse, an infinitely greater misfortune. Debt blunts and blights the finest sensibilities of our nature; it eats out the sweetest domestic affections; it blasts the moral character; it robs us of our manliness, and where there was once all that was noble, truthful, high-minded, there is nothing left but the charred waste of debased manhood, of contemptible prevarications, and mean concealments. The Demon of Debt! how it withers and wilts the beautiful flowers of conjugal love, of parental affection, and the holier emotions that belong to the Infinite One! How it poisons every gladness, robs every smile of its beauty, cuts up by the root every glorious quality of our nature, and makes of him who might have been a man, a poor, fawning, faltering, cringing wretch, waiting the creditor's utterances with the fears of a slave, with the trembling of a culprit; the fire has no warmth, the food no taste, the flower no beauty, the air no life, the sky no sun; the brain perceives nothing, the eye sees nothing, the heart feels nothing but the chill damps of the specter Debt, in the person of the creditor, that so looms up in the daytime as to shut out all the blue sky of life, and in the hours of sleep, sits like a horrid nightmare, with the weight of Pelion on Olympus piled. With these before him, who but an idiot could be induced to incur an indebtedness which there was not ample means on hand to satisfy, if necessary, within the hour?

